# Pharmacokinetics and safety of SHR0302, a selective JAK1 inhibitor, in Chinese patients with hepatic impairment

**DOI:** 10.3389/fphar.2026.1857187

**Published:** 2026-07-02

**Authors:** Jiajia Mai, Hongda Lin, Min Wu, Di Wang, Hong Zhang, Qinglong Jin, Chong Chen

**Affiliations:** 1 Department of Phase I Clinical Trial Unit, The First Hospital of Jilin University, Changchun, Jilin, China; 2 Clinical Pharmacology Department, Jiangsu Hengrui Pharmaceuticals Co., Ltd., Jiangsu, China; 3 Department of Hepatology, Center of Infectious Diseases and Pathogen Biology, The First Hospital of Jilin University, Changchun, China; 4 Abdominal Ultrasound Department, The First Hospital of Jilin University, Changchun, Jilin, China

**Keywords:** clinical trial, hepatic impairment, JAK1 inhibitor, pharmacokinetics, SHR0302

## Abstract

**Objective:**

To evaluate the effects of mild and moderate hepatic impairment on the pharmacokinetics and safety of SHR0302.

**Methods:**

This open-label, parallel-group study enrolled 24 Chinese subjects, including subjects with normal hepatic function and those with mild or moderate hepatic impairment (8 per group). All subjects received a single oral dose of SHR0302 (8 mg). Plasma PK parameters of SHR0302 and its metabolite, SHR161279, were assessed and compared across groups. Safety was evaluated throughout the study.

**Results:**

Mild hepatic impairment had minimal effect on the exposure of SHR0302. In subjects with moderate hepatic impairment, the C_max_ of SHR0302 was approximately 17% lower than that in subjects with normal hepatic function, whereas AUC_0-t_ and ${\text{AUC}}_{0‐\infty }$ remained generally unchanged. Meanwhile, exposure to SHR161279 decreased in both hepatic impairment groups, with reductions of approximately 21%–38%. SHR0302 was generally safe after single-dose administration. Eight subjects (8/24, 33.3%) experienced treatment-emergent adverse events (TEAEs). No serious adverse events were reported.

**Conclusion:**

Mild and moderate hepatic impairment had minimal effect on SHR0302 exposure. Based on the single-dose pharmacokinetic and safety data, dose adjustment of SHR0302 may not be necessary in patients with mild or moderate hepatic impairment.

**Clinical Trial Registration:**

https://clinicaltrials.gov/, identifier NCT04293029.

## 1 Introduction

Janus kinases (JAKs) belong to the family of non-receptor tyrosine kinases, serve as important intracellular mediators of signal transduction by multiple cytokine and interferon receptors (Schindler and Plumlee, 2008; Glassman et al., 2022). The JAK/STAT signaling pathway plays a central role in inflammatory responses and immune regulation. Upon binding of cytokines to cell surface receptors, the associated JAKs are activated and subsequently phosphorylate signal transducers and activators of transcription (STATs), promoting their dimerization and nuclear translocation, where they bind to regulatory regions of target genes and modulate downstream gene transcription (Darnell, 1997). This pathway is critically involved in the pathogenesis of multiple immune-mediated inflammatory diseases. Oral small-molecule JAK inhibitors targeting the JAK/STAT pathway have therefore become an important therapeutic strategy. They have been used clinically in rheumatic diseases (van Vollenhoven et al., 2012), inflammatory bowel disease (Sandborn et al., 2017), and immune-mediated skin disorders (Silverberg et al., 2020).

The JAK family comprises four members: JAK1, JAK2, JAK3, and tyrosine kinase 2 (TYK2) (Yamaoka et al., 2004). Among these, JAK1 occupies a key position in the signaling of multiple pro-inflammatory cytokines. It is involved in the mediation of several inflammation-related cytokine pathways, including common γ-chain cytokines such as interleukin (IL)-2, IL-4, IL-7, IL-9, IL-15, and IL-21 (Yamaoka et al., 2004), as well as pro-inflammatory cytokines such as IL-6 and interferon (IFN)-related signaling pathways (Spinelli et al., 2021). In theory, selective inhibition of JAK1 may preserve anti-inflammatory effects while reducing interference with physiological signaling pathways mediated by other JAK isoforms, thereby potentially improving treatment safety. Accordingly, it has become one of the major directions in the development of JAK inhibitors in recent years. SHR0302 tablets (SHR0302) is a JAK inhibitor targeting JAK1. The drug was jointly developed by Jiangsu Hengrui Pharmaceuticals Co., Ltd. and its subsidiaries. In 2015, it received approval from the former National Medical Products Administration (NMPA) to enter clinical trials as a Class 1.1 innovative chemical drug in China.

The liver is an important organ for drug metabolism and elimination. It mediates drug metabolism through multiple biotransformation pathways, including oxidation, reduction, hydrolysis, and conjugation, and contributes to the elimination of drugs and their metabolites through biliary excretion. Hepatic impairment may alter drug metabolism and excretion, thereby leading to the accumulation of drugs or their metabolites in the body. In some cases, it may also reduce the formation of active metabolites and consequently affect drug efficacy. A human mass balance study showed that, following oral administration of SHR0302 under fasting conditions, only a small proportion of the parent drug was excreted unchanged in urine and feces, with cumulative excretion rates of 2.796% and 9.468%, respectively, indicating that SHR0302 is eliminated predominantly through metabolism. The cumulative excretion rates of its mono-oxidized metabolite, SHR161279, were 26.319% in urine and 2.785% in feces, with a total exceeding 20%. The US Food and Drug Administration (FDA) recommends that pharmacokinetic studies in subjects with hepatic impairment should generally be conducted for drugs in which hepatic metabolism and/or biliary excretion accounts for more than 20% of the absorbed dose (U.S. Food and Drug Administration, 2003). Accordingly, this study was conducted to evaluate the pharmacokinetic characteristics, safety, and tolerability of SHR0302 in Chinese subjects with mild and moderate hepatic impairment (Child-Pugh class A and B). Expected to provide evidence for the rational clinical use and dose recommendation of SHR0302 in patients with hepatic impairment. Based on previous *in vitro* plasma protein binding studies, the plasma protein binding rates of SHR0302 in human plasma were 50.2% ± 3.2%, 48.5% ± 0.7%, and 42.4% ± 0.1% at concentrations of 200, 1,000, and 5,000 ng/mL, respectively, corresponding to estimated unbound fractions of approximately 49.8%, 51.5%, and 57.6%. These values are substantially higher than the threshold for highly protein-bound drugs defined in the FDA guidance on hepatic impairment pharmacokinetic studies, in which a fraction unbound <10% is considered extensive plasma protein binding and may warrant measurement of the unbound fraction in drugs with a high hepatic extraction ratio. Therefore, SHR0302 is not considered a highly plasma protein-bound drug, and hepatic impairment is unlikely to markedly affect unbound SHR0302 exposure through altered protein binding. Accordingly, unbound SHR0302 concentrations or the unbound fraction were not measured in participants with hepatic impairment in this study.

## 2 Materials and methods

### 2.1 Ethical approval

This study protocol was approved by the Ethics Committee of the Clinical Research Institute, the First Hospital of Jilin University (Changchun, China, approval numbe: 20Y004-001). All procedures were conducted in accordance with the standards of the Phase I Clinical Trial Unit, the Declaration of Helsinki, and Good Clinical Practice (GCP) guidelines. Written informed consent was obtained from all participants prior to enrollment.

### 2.2 Subjects

Volunteers aged 18–65 years old, with body weight ≥50 kg for males and ≥45 kg for females, and a body mass index (BMI) between 18 and 30 kg/m^2^ were enrolled in this trial. The main Exclusion Criteria for all the subjects included: 1) Clinically significant infection requiring systemic antimicrobial therapy within 4 weeks prior to screening, including evidence/history of active or latent tuberculosis, or a history of recurrent/disseminated herpes zoster or disseminated herpes simplex. 2) Use of JAK inhibitors within 3 months prior to dosing. 3) Concomitant medications or products likely to affect SHR0302 pharmacokinetics (including strong CYP3A4 inhibitors/inducers, prescription/OTC drugs, herbal products, or supplements) within 2 weeks prior to screening. 4) Clinically significant comorbidities or investigator-judged conditions that could increase risk or interfere with study completion (e.g., serious cardiovascular, neuropsychiatric, respiratory, renal, or endocrine disease), including malignancy within 5 years 5) Hypertension (resting SBP ≥160 mmHg and/or DBP ≥100 mmHg) or clinically significant ECG abnormalities (e.g., QTc ≥450 ms, second-/third-degree AV block, or arrhythmias requiring treatment). 6) Clinically relevant laboratory abnormalities at screening, including WBC <3.0 × 10^9^/L and/or ANC <1.5 × 10^9^/L, eGFR <90 mL/min, or HbA1c ≥ 8%. 7) Substance abuse or a positive urine drug screen; or alcohol use inconsistent with protocol restrictions. 8) Major surgery likely to affect drug absorption (e.g., major gastric resection) or major surgery within 6 months prior to screening/anticipated during the study. 9) Participation in another interventional clinical trial within 1 month prior to dosing (or within 5 half-lives of the investigational product).

Hepatic impairment patients were required to meet the following criteria:1). Stable treatment for at least 4 weeks before study drug administration, or no medication use during this period; 2) hepatic dysfunction secondary to primary liver diseases (e.g., hepatitis B, hepatitis C, autoimmune hepatitis), classified as Child-Pugh class A or B; and the exclusion Criteria included:1) history of liver transplantation, hepatic failure, or cirrhosis with severe complications (e.g., hepatic encephalopathy, hepatocellular carcinoma, esophagogastric variceal bleeding).

Normal hepatic function subjects were enrolled as matched controls, with matching criteria including age (±10 years), gender, and body weight (±10 kg).

### 2.3 Study design and administration

**FIGURE 1:**
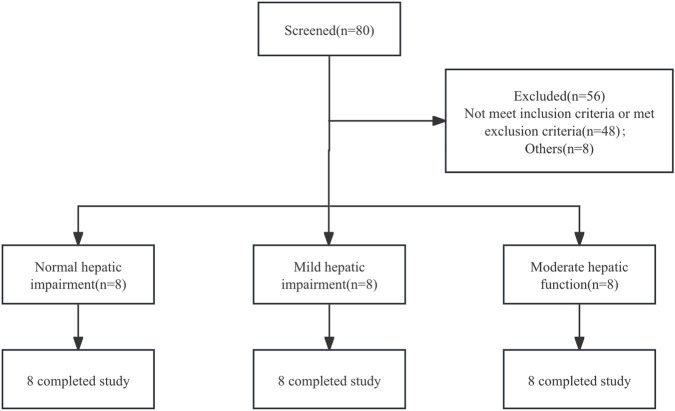
Study design flowchart.

This was a phase I, open-label, parallel-design trial. In accordance with FDA guidance for reduced hepatic impairment pharmacokinetic study designs, which recommends at least eight evaluable subjects in both the normal hepatic function and moderate hepatic impairment groups, eight subjects were enrolled in each group in the present study (U.S. Food and Drug Administration, 2003). A total of 24 subjects were enrolled and divided into three groups: mild hepatic impairment (Child-Pugh class A, score 5–6), moderate hepatic impairment (Child-Pugh class B, score 7–9), and healthy controls, with 8 subjects in each group. The participant flow was shown in Figure 1. Eligible subjects were admitted to the clinical research center on Day-1 (D-1). Subjects received a single oral dose of SHR0302 tablets (8 mg) on Day 1 (D1) after an overnight fast of at least 10 h. Water intake was prohibited for 1 h before and 1 h after dosing, and food was prohibited for 4 h after dosing. Subjects were discharged from the study center on Day 4 (D4) after complet the study assessments.

### 2.4 Safety assessment

Safety assessments were performed in accordance with internationally recognized standards. Adverse events (AEs) were monitored throughout the study to evaluate their frequency and severity. Safety evaluation included vital signs (body temperature, heart rate, and blood pressure), physical examinations, 12-lead ECGs, and clinical laboratory tests. Laboratory assessments comprised hematology, serum biochemistry, urinalysis, and coagulation parameters.

### 2.5 Pharmacokinetic and pharmacodynamic sample collection and analysis

For PK evaluation, approximately 4 mL of blood was collected into each EDTA-K2 tube within 1 h before dosing and at 0.25, 0.5, 1, 1.5, 2, 3, 4, 6, 8, 10, 12, 24, 48, 72 h post-dose for the determination of plasma SHR0302 and its metabolite (SHR161279) concentrations. EDTA-K2 tubes were inverted 5 to 10 times, and centrifuged at 2000 *g* for 10 min at 2 °C–8 °C within 0.5 h of collection. Then the separated plasma were stored at −80 °C within 2 h. Plasma concentrations of SHR0302 and SHR161279 were quantified using a validated LC–MS/MS method by Shanghai Funda Biotech Co., Ltd., in accordance with the company’s standard operating procedures.

Calibration curves for both analytes were linear over 1–1,000 ng/mL, with a lower limit of quantitation (LLOQ) of 1 ng/mL. At the LLOQ level, intra- and inter-batch precision (%CV) were ≤8.3% and ≤6.2% for SHR0302 and ≤8.1% and ≤9.0% for SHR161279, respectively. Accuracy (bias%) at the LLOQ level ranged from −4.2% to 5.1% (intra-batch) and was 0.6% (inter-batch) for SHR0302, and from −3.1% to 9.0% (intra-batch) and 1.4% (inter-batch) for SHR161279.

Precision and accuracy for quality-control samples met established acceptance criteria for bioanalytical methods. Both analytes were stable in plasma for 21 h at room temperature, over five freeze-thaw cycles (−70 °C to room temperature), and for up to 294 days when stored at −20 °C or −70 °C.

### 2.6 Statistical analysis

Pharmacokinetic (PK) parameters were calculated by noncompartmental analysis (NCA) using Phoenix WinNonlin software (version 8.0; Pharsight Certara, United States). PK parameters included maximum observed concentration (C_max_), time to reach Cmax (T_max_), area under the concentration-time curve from time zero to the last measurable concentration (AUC_0-t_), area under the curve extrapolated to infinity ( ${\text{AUC}}_{0‐\infty }$ ), terminal elimination half-life (t_1/2_), apparent clearance (CL/F), and apparent volume of distribution (Vz/F).

Calculation of PK parameter ratios. For the primary pharmacokinetic parameters (C_max_, AUC_0-t_, and ${\text{AUC}}_{0‐\infty }$ ), values were natural log-transformed and analyzed using an analysis of variance (ANOVA) model with hepatic function group as a fixed effect. Least-squares mean differences between each hepatic impairment group (mild or moderate) and the normal hepatic function group, together with the corresponding 90% confidence intervals (CIs), were estimated on the log scale. These estimates were exponentiated to obtain the geometric mean ratios (hepatic impairment/normal) and their corresponding 90% CIs on the original scale.

## 3 Results

### 3.1 Demographics of the subjects

24 Chinese participants were enrolled and received study drug: including 8 with normal hepatic function, 8 with mild hepatic impairment, and 8 with moderate hepatic impairment. Baseline demographic characteristics are presented in Table 1.

**TABLE 1:** Demographic characteristics of three groups.

Characteristics (Units)	Normal (n = 8)	Mild hepatic impairment (n = 8)	Moderate hepatic impairment (n = 8)
Age (y), mean (SD)	44.5 (4.21)	44.4 (5.40)	52.4 (5.42)
Male, n (%)	6 (75.0)	7 (87.5)	6 (75.0)
Weight (kg), mean (SD)	67.65 (4.304)	70.83 (9.936)	70.09 (10.097)
Height (cm), mean (SD)	162.74 (7.975)	169.71 (9.013)	163.33 (11.825)
BMI (kg/m^2^), mean (SD)	25.66 (2.533)	24.55 (2.655)	26.18 (1.325)
Child–Pugh score, mean (SD)	NA	5.00 (0.000)	7.75 (0.463)

Abbreviations: *y*, years; n, number; BMI, body mass index; Data are presented as the mean (SD), and categorical variables are presented as frequency (percentage).

### 3.2 Safety

No deaths or serious adverse events (SAEs) were reported during the trial. No subjects withdrew prematurely, and all enrolled subjects were included in the safety analysis.

Among the 24 subjects, 8 subjects (8/24, 33.3%) experienced treatment-emergent adverse events (TEAEs). In the mild hepatic impairment group, 2 of 8 subjects (2/8, 25%) reported TEAEs, all of which were mild. In the moderate hepatic impairment group, 6 of 8 subjects (6/8, 75.0%) reported TEAEs, all TEAEs were mild except for one event of moderate severity. No TEAEs were reported in the normal liver function group. The most common TEAEs (incidence ≥5%) were decreased platelet count (3/24, 12.5%), decreased neutrophil count (3/24, 12.5%), decreased white blood cell count (2/24, 8.3%), decreased lymphocyte count (2/24, 8.3%), decreased serum albumin (2/24, 8.3%) (Table 2 **)**.

**TABLE 2:** TEAEs in three groups (incidence rate ≥5%).

TEAEs	Normal (n = 8)	Mild hepatic impairment (n = 8)	Moderate hepatic impairment (n = 8)
Platelet count decreased	0	1 (12.5%)	2 (25%)
Decreased neutrophil count	0	1 (12.5%)	1 (12.5%)
White blood cell count decreased	0	0	2 (25%)
Lymphocyte count decreased	0	0	2 (25%)
Decreased serum albumin	0	0	2 (25%)

Data are reported as *n* (%). Abbreviations: *n*, number of TEAEs; *n* (%), incidence of subjects reporting TEAEs.

### 3.3 PK of SHR0302 and SHR161279

**FIGURE 2:**
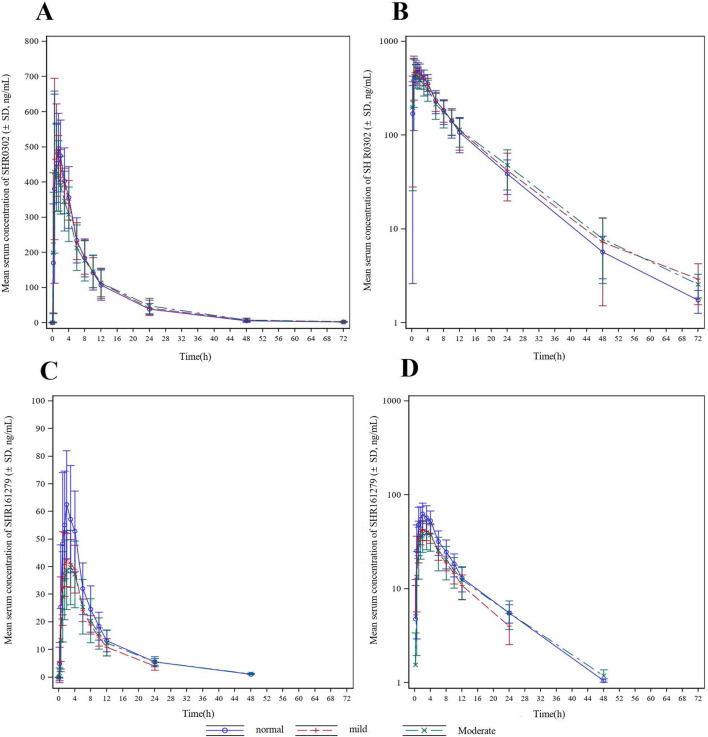
Time profiles of mean plasma concentration of SHR0302 and SHR161279 in pharmacokinetic analysis set. **(A)** Mean plasma concentration–time profile of SHR0302. **(B)** Mean plasma concentration–time profile of SHR0302 (semi-logarithmic scale). **(C)** Mean plasma concentration–time profile of SHR161279. **(D)** Mean plasma concentration–time profile of SHR161279 (semi-logarithmic scale).

Following administration of a single 8 mg oral dose of SHR0302, mean plasma concentration–time profiles of SHR0302 are shown in Figure 2A (linear scale) and Figure 2B (semi-log scale) for subjects with normal hepatic function, mild hepatic impairment, and moderate hepatic impairment. Mean concentration-time profiles of the metabolite SHR161279 are shown in Figure 2C (linear scale) and Figure 2D (semi-log scale) for the same groups.

The geometric mean (GCV%) AUC_0-t_, and ${\text{AUC}}_{0‐\infty }$ , Cmax, of plasma SHR0302 were 4,290 h ng/mL (27.8%), and 4,330 h ng/mL (27.6%), 580 ng/mL (13.4%) in subjects with normal hepatic function; 4,440 h ng/mL (27.2%), and 4,480 h ng/mL (27.3%), 563 ng/mL (13.5%) in those with mild impairment; and 4,240 ng/mL (34.1%), 4,270 h ng/mL (34.1%), and 482 h ng/mL (31.4%) in subjects with moderate hepatic function. For metabolite SHR161279, the corresponding values were 520 h ng/mL (27.4%), and 568 h ng/mL (22.4%), 61.8 ng/mL (30.1%) in normal hepatic function; 382 h ng/mL (15.6%), and 427 h ng/mL (15.5%), 42.8 ng/mL (25.8%) in mild impairment; and 402 h ng/mL (37.3%), and 450 h ng/mL (35%), 38.4 ng/mL (40.9%) in moderate hepatic function (Table 3).

**TABLE 3:** PK parameters of SHR0302 and SHR161279.

Analyte	PK parameter	Normal (n = 8)	Mild hepatic impairment (n = 8)	Moderate hepatic impairment (n = 8)
SHR0302	AUC_0-t_ (h*ng/mL)	4,290 (27.8)	4,440 (27.2)	4,240 (34.1)
	${\text{AUC}}_{0‐\infty }$ (h*ng/mL)	4,330 (27.6)	4,480 (27.3)	4,270 (34.1)
	Cmax (ng/mL)	580 (13.4)	563 (13.5)	482 (31.4)
	CL/f (mL/h)	1850 (27.6)	1790 (27.3)	1870 (34.1)
	Tmax(h)	0.75 (0.50, 2.00)	0.5 (0.50, 1.50)	0.5 (0.50, 2.00)
	t_1/2_(h)	8.64 (1.03)	8.99 (1.75)	9.08 (1.65)
	Vz/f (mL)	22,900 (23.5)	22,800 (14.5)	24,100 (22.9)
SHR161279	AUC_0-t_ (h*ng/mL)	520 (27.4)	382 (15.6)	402 (37.3)
	${\text{AUC}}_{0‐\infty }$ (h*ng/mL)	568 (22.4)	427 (15.5)	450 (35)
	Cmax (ng/mL)	61.8 (30.1)	42.8 (25.8)	38.4 (40.9)
	Tmax(h)	2 (1.00, 3.00)	2.5 (1.50, 4.00)	3 (2.00, 4.00)
	t_1/2_(h)	8.52 (1.73)	7.62 (1.48)	9.57 (2.17)

Data are shown as geometric mean (geometric coefficient of variation, %), except for T_max_, which is expressed as median (minimum-maximum),and t_1/2_ expressed as mean value ±SD.

ANOVA model analysis showed that, compared with normal hepatic function, the geometric mean ratios (GMRs) and corresponding 90% CIs of plasma SHR0302 were 0.972 (0.813, 1.162) and 0.832 (0.696, 0.995) for Cmax, 1.036 (0.806, 1.332) and 0.988 (0.769, 1.270) for AUC_0-t,_ and 1.035 (0.805, 1.330) and 0.987 (0.768, 1.268) for ${\text{AUC}}_{0‐\infty }$ in mild and moderate hepatic impairment groups, respectively. For plasma SHR161279, the GMRs (90% CI) were 0.693 (0.526, 0.912) and 0.621 (0.472, 0.817) for Cmax, 0.734 (0.579, 0.929) and 0.773 (0.610, 0.980) for AUC_0-t_, and 0.752 (0.606, 0.933) and 0.792 (0.638, 0.983) for ${\text{AUC}}_{0‐\infty }$ , respectively (Table 4).

**TABLE 4:** Ratios for PK of SHR0302 and SHR161279 in subjects with hepatic impairment versus normal hepatic function.

Analyte	Parameter	Mild hepatic impairment (n = 8)	Moderate hepatic impairment (n = 8)
SHR0302	Cmax	0.972 (0.813, 1.162)	0.832 (0.696, 0.995)
	AUC_0-t_	1.036 (0.806, 1.332)	0.988 (0.769, 1.270)
	${\text{AUC}}_{0‐\infty }$	1.035 (0.805, 1.330)	0.987 (0.768, 1.268)
SHR161279	Cmax	0.693 (0.526, 0.912)	0.621 (0.472, 0.817)
	AUC_0-t_	0.734 (0.579, 0.929)	0.773 (0.610, 0.980)
	${\text{AUC}}_{0‐\infty }$	0.752 (0.606, 0.933)	0.792 (0.638, 0.983)

Data are shown as least-squares geometric mean ratios (90% confidence intervals).

## 4 Discussion

This study assessed the effects of mild and moderate hepatic impairment on the pharmacokinetics of SHR0302 and its metabolite SHR161279, and further evaluated the safety of SHR0302 in these patients. Mild hepatic impairment had minimal effect on the exposure of SHR0302. In subjects with moderate hepatic impairment, Cmax was approximately 17% lower than that in subjects with normal hepatic function, whereas AUC remained generally unchanged. Overall, these findings suggest that mild or moderate hepatic impairment has only a limited impact on the overall pharmacokinetics of SHR0302. The lower Cmax observed in the moderate hepatic impairment group may be attributable to intersubject variability.

Compared with subjects with normal hepatic function, those with mild hepatic impairment had lower exposure to the metabolite SHR161279, with Cmax, AUC0-t, and AUC0‐∞ decreasing by approximately 31%, 27%, and 25%, respectively. A similar trend was observed in subjects with moderate hepatic impairment, in whom the corresponding decreases were approximately 38%, 23%, and 21%. Overall, exposure to SHR161279 was reduced in both the mild and moderate hepatic impairment groups. *In vitro* studies using recombinant human enzymes showed that the major metabolic pathways of SHR0302 were N-demethylation and mono-oxidation. Multiple CYP450 enzymes were involved in its metabolism, with CYP3A4 and CYP1B1 making the greatest contribution. In recombinant human CYP450 incubation systems, the major detected metabolites were the N-demethylated metabolite M1 and the mono-oxidized metabolite M3. Based on the relative hepatic abundance and catalytic activity of CYP450 enzymes, the formation of M3 (SHR161279) was considered to be mainly mediated by CYP3A4, CYP1B1, and CYP2D6. In our *in vivo* PK analysis, exposure to SHR161279 decreased by approximately 21%–38% in subjects with hepatic impairment. Mechanistically, this reduction may be related to decreased formation of the metabolite under impaired hepatic function. However, despite the reduced exposure to SHR161279, this change is unlikely to have a meaningful impact on the overall efficacy or safety of SHR0302. SHR161279 accounts for only about 12% of the exposure of the parent drug and shows weaker *in vitro* activity than SHR0302. Its contribution to the overall pharmacological effect is therefore expected to be limited. Accordingly, dose adjustment does not appear to be necessary in subjects with mild or moderate hepatic impairment.

In this study, 8 of the 24 enrolled subjects (8/24, 33.3%) experienced TEAEs. No subject withdrew because of an adverse event, and no serious adverse events (SAEs) occurred. The incidence of TEAEs was numerically higher in participants with moderate hepatic impairment than in those with mild hepatic impairment; however, this finding should be interpreted cautiously. As SHR0302 is a JAK1 inhibitor, hematologic safety is clinically relevant because the JAK-STAT pathway participates not only in inflammatory and immune signaling but also in hematopoietic stem/progenitor cell maintenance and blood cell differentiation. Consistently, hematologic abnormalities have been reported with approved JAK1 or relatively selective JAK1 inhibitors, including upadacitinib and abrocitinib, for which complete blood count monitoring is recommended before and during treatment (AbbVie Inc, 2026; Pfizer Inc, 2023). In our study, the higher TEAE incidence in the moderate hepatic impairment group was not accompanied by increased exposure to SHR0302 or SHR161279: SHR0302 C_max_ was approximately 17% lower, whereas AUC_0-t_ and ${\text{AUC}}_{0‐\infty }$ were not meaningfully different, and SHR161279 C_max_, AUC_0-t_, and ${\text{AUC}}_{0‐\infty }$ were decreased by approximately 38%, 23%, and 21%, respectively, compared with subjects with normal hepatic function. These PK findings do not support an exposure-driven explanation for the higher TEAE incidence. Moreover, participants with Child-Pugh class B hepatic impairment generally have reduced hepatic functional reserve and compensatory capacity, and laboratory parameters may fluctuate because of underlying chronic liver disease, short-term physiological stress, or other nonspecific factors. Therefore, this numerical imbalance should be interpreted in light of the small sample size, single-dose design, and underlying disease status, while complete blood count monitoring remains important in future multiple-dose studies and clinical practice.

Our study provides clinically relevant evidence regarding the use of SHR0302 in patients with hepatic impairment. Nevertheless, some limitations should be noted. First, only subjects with Child–Pugh class A or B hepatic impairment were included; therefore, the findings cannot be extended to patients with severe hepatic impairment (Child–Pugh class C). Second, because the study population consisted exclusively of Chinese participants, the applicability of these results to other ethnic groups remains to be established.

## 5 Conclusion

Mild to moderate hepatic impairment had minimal effect on the exposure of SHR0302, although exposure to its metabolite SHR161279 was slightly reduced. Based on the single-dose pharmacokinetic and safety data, dose adjustment of SHR0302 may not be necessary in patients with mild or moderate hepatic impairment. SHR0302 was generally safe and well tolerated after a single oral dose in subjects with normal hepatic function and in those with mild or moderate hepatic impairment. As the incidence of adverse events tended to increase with worsening hepatic impairment, careful monitoring of hematologic and hepatic parameters is warranted in these patients.

## Data Availability

The original contributions presented in the study are included in the article/supplementary material, further inquiries can be directed to the corresponding authors.
